# Different but overlapping populations of *Strongyloides stercoralis* in dogs and humans—Dogs as a possible source for zoonotic strongyloidiasis

**DOI:** 10.1371/journal.pntd.0005752

**Published:** 2017-08-09

**Authors:** Tegegn G. Jaleta, Siyu Zhou, Felix M. Bemm, Fabian Schär, Virak Khieu, Sinuon Muth, Peter Odermatt, James B. Lok, Adrian Streit

**Affiliations:** 1 Department of Evolutionary Biology, Max-Planck-Institute for Developmental Biology, Tübingen, Germany; 2 Department of Pathobiology, School of Veterinary Medicine, University of Pennsylvania, Philadelphia, Pennsylvania, United States of America; 3 Department of Molecular Biology, Max-Planck-Institute for Developmental Biology, Tübingen, Germany; 4 Department of Epidemiology and Public Health, Swiss Tropical and Public Health Institute, Basel, Switzerland; 5 University of Basel, Basel, Switzerland; 6 National Center for Parasitology, Entomology and Malaria Control, Ministry of Health, Phnom Penh, Cambodia; Vienna, AUSTRIA

## Abstract

Strongyloidiasis is a much-neglected soil born helminthiasis caused by the nematode *Strongyloides stercoralis*. Human derived *S*. *stercoralis* can be maintained in dogs in the laboratory and this parasite has been reported to also occur in dogs in the wild. Some authors have considered strongyloidiasis a zoonotic disease while others have argued that the two hosts carry host specialized populations of *S*. *stercoralis* and that dogs play a minor role, if any, as a reservoir for zoonotic *S*. *stercoralis* infections of humans. We isolated *S*. *stercoralis* from humans and their dogs in rural villages in northern Cambodia, a region with a high incidence of strongyloidiasis, and compared the worms derived from these two host species using nuclear and mitochondrial DNA sequence polymorphisms. We found that in dogs there exist two populations of *S*. *stercoralis*, which are clearly separated from each other genetically based on the nuclear 18S rDNA, the mitochondrial *cox*1 locus and whole genome sequence. One population, to which the majority of the worms belong, appears to be restricted to dogs. The other population is indistinguishable from the population of *S*. *stercoralis* isolated from humans. Consistent with earlier studies, we found multiple sequence variants of the hypervariable region I of the 18 S rDNA in *S*. *stercoralis* from humans. However, comparison of mitochondrial sequences and whole genome analysis suggest that these different 18S variants do not represent multiple genetically isolated subpopulations among the worms isolated from humans. We also investigated the mode of reproduction of the free-living generations of laboratory and wild isolates of *S*. *stercoralis*. Contrary to earlier literature on *S*. *stercoralis* but similar to other species of *Strongyloides*, we found clear evidence of sexual reproduction. Overall, our results show that dogs carry two populations, possibly different species of *Strongyloides*. One population appears to be dog specific but the other one is shared with humans. This argues for the strong potential of dogs as reservoirs for zoonotic transmission of *S*. *stercoralis* to humans and suggests that in order to reduce the exposure of humans to infective *S*. *stercoralis* larvae, dogs should be treated for the infection along with their owners.

## Author summary

Infections of humans with the nematode *Strongyloides stercoralis* can persist for a very long time, due to the capacity of this pathogen to undergo an autoinfective life cycle and re-infect the same host over and over again. Clinical manifestation, known as human stongyloidiasis, may be fatal and can arise many years and generations of worms after the initial infection occurred. Although *Strongyloides stercoralis* has been known as the causative agent of strongyloidiasis for a very long time, some key questions about its biology and epidemiology remain open. Here we address two of them. Firstly, it has long been known that dogs can serve as experimental hosts for *S*. *stercoralis* but it is a matter of debate whether *Strongyloides* spp. found in dogs in the wild are human pathogenic *S*. *stercoralis*, and whether dogs therefore are a source of zoonotic transmission of this parasite. Here we show that dogs carry two genetically different populations of *Strongyloides* spp. one of which is shared with humans. This demonstrates that dogs represent a possible reservoir for zoonotic strongyloidiasis. Secondly, the all female, parthenogenetic parasitic generations may alternate with single facultative free-living generations, which consist of both females and males. In spite of the presence of both sexes, it had been postulated that males do not contribute genetic material to the progeny and that sperm are merely required to trigger parthenogenetic embryonic development. Here we show that the free-living adults of *S*. *stercoralis* reproduce sexually.

## Introduction

**Fig 1:**
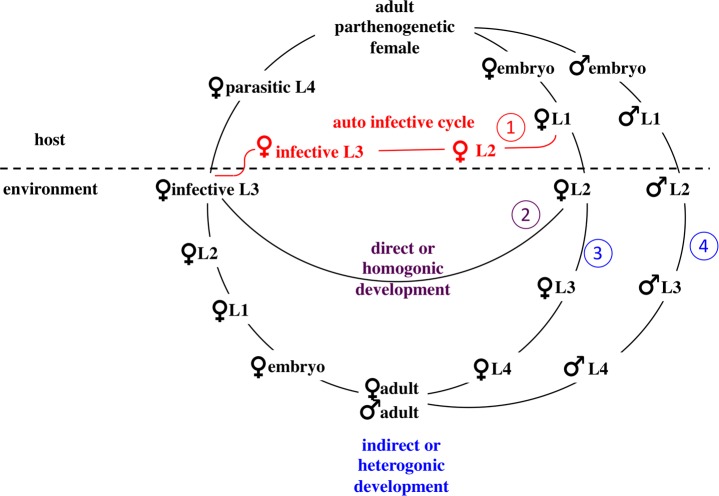
The life cycle of *S*. *stercoralis*. The life cycle of *Strongyloides stercoralis*. The numbers refer to the numbers of the developmental options in the description of the life cycle in the text.

Soil-transmitted helminthiasis (STH) affects up to one in four individuals in the world, disproportionately impacting impoverished populations with less access to clean water, sanitation, and opportunities for socioeconomic development [1]. Strongyloidiasis is one of the most neglected tropical diseases [2,3]. Estimates of the number of people infected with the causative agent *Strongyloides stercoralis* vary and go up to 370 million worldwide [2,4,5]. The local prevalence can reach more than 40% in some tropical and subtropical countries [3,6]. Factors such as high temperature, high moisture, poor sanitation and sharing premises with domestic animals may contribute to high prevalence of *S*. *stercoralis* [3,7,8]. *S*. *stercoralis* is the major causative agent of human strongyloidiasis [9] but there are also reports of people infected with *Strongyloides fuelleborni* and *Strongyloides fuelleborni kellyi*, in Africa and in Papua New Guinea [9]. Based on molecular data, *S*. *fuelleborni kellyi* should probably be considered an independent species rather than a subspecies of *S*. *fuelleborni*, [10]. Although *S*. *stercoralis* infection frequently remains asymptomatic, immuno-compromised patients can develop a systemic infection, which may lead to fatal forms of strongyloidiasis. The medical relevance of this parasite has probably been grossly underestimated due to difficulty of diagnosis [4,5,11]. Also, it should be noted that *Strongyloides* is not limited to tropical and underdeveloped areas, and the presence of *S*. *stercoralis* and fatal cases caused by it have also been reported from well-developed regions with temperate climates such as the European Union and North America [12–18]. *S*. *stercoralis* has a complex, rather unique life cycle (Fig 1) consisting of parasitic and free-living generations [19–21]. In brief: infective third stage larvae (L3i), which are all females, invade a new host by skin penetration and, after migrating through the blood and the lungs, are coughed up and swallowed and eventually establish in the small intestine of the host. The parasitic adult females reproduce by parthenogenesis. The progeny of the parasitic females have four developmental options: 1) Firstly, they may become female, and develop into infective third stage larvae (iL3) within the host and re-infect the same host individual (autoinfective cycle); 2) Secondly, they may become female, but this time leave the host as first-stage larvae, develop into iL3 and search for a new host (direct/homogonic development); 3) Thirdly, they may become female and leave the host, but this time develop into free-living, non-infective third stage larvae and subsequently into adult females (indirect/heterogonic development); 4) Or fourthly, they become male and leave the host and develop into free-living adult males (indirect/heterogonic cycle). The free-living adults mate and reproduce in the environment and all their progeny are females and develop to iL3s. No male iL3s have been reported in any *Strongyloides* species. For two species of *Strongyloides* (*Strongyloides ratti* and *Strongyloides papillosus*), it has been shown that the reproduction in the free-living generation is sexual, in spite of some earlier literature that had described it as pseudogamic (by sperm dependent parthenogenesis) [22–24]. For *S*. *stercoralis* prior to this report no genetic analysis of the mode of reproduction had been conducted and non-sexual (pseudogamic) reproduction as proposed based on cytological observations remained an option [25,26]. Whilst all species of *Strongyloides* may undergo homogonic or heterogonic development, the autoinfective cycle (option 1) appears to be specific for *S*. *stercoralis* and maybe a few other less well-investigated species [19]. This autoinfective cycle allows the parasite to persist in a particular host individual for many years, much longer than the life expectancy of an individual worm. Usually, healthy individuals tolerate such long lasting infections well and control them at very low levels [5]. These people have no clinical symptoms and the infection is unlikely to be detected. However, if such a chronically infected person becomes immunodeficient due to disease or immunosuppressive treatment (i.e. cancer chemotherapy or organ transplantation), this may lead to failure to control the infection and consequentially to self-enhancing progression of strongyloidiasis (hyperinfection syndrome and disseminated strongyloidiasis), which is lethal if not treated [5].

Parts of the 18S rDNA (small Subunit, *SSU*) sequence, in particular the hypervariable regions (HVR) I and IV, are widely used as nuclear markers for molecular taxonomy of nematodes in general (e.g. [27–33]) and *Strongyloides* spp. in particular [8,34–36]. Whilst some sequence variation in HVR I within *S*. *stercoralis* was reported [35,37], HVR IV appears virtually invariable within this species. To our knowledge, there is only one report to date of a single nucleotide difference within this region [38](accession number M84229).

Whilst humans are their natural hosts, dogs, cats, and non-human primates have also been proposed to be suitable hosts for *S*. *stercoralis* [39–42]. To what extent strongyloidiasis is a zoonotic disease has been the subject of controversy in the literature for several decades. Originally Brumpt (1922) [43], later supported by Augustine (1940) [44] split the *Strongyloides* of dogs from *S*. *stercoralis* and described it as a separate species, called *Strongyloides canis*. Recent comparative analyses of the mitochondrial locus cytochrome c oxidase subunit 1 (*cox*1) [6,36,38,45] and the whole genome sequence of 33 individual *S*. *stercoralis* from Japan and Myanmar [46] indicated that there is substantial genetic diversity among *S*. *stercoralis* isolated from human hosts and [38] suggested that there might exist human- and dog-specialized subpopulations. On the other hand, dogs have long been known to be suitable experimental hosts for human derived *S*. *stercoralis* [39,41], and many authors consider *Strongyloides* in dogs and humans to belong to the same species, i.e. *S*. *stercoralis*. While the more recent literature appears to favor separation, it remains unclear whether *S*. *stercoralis* naturally infecting dogs and humans belong to the same populations or not, and correspondingly, what the potential is for dogs to serve as a source for human *S*. *stercoralis* infections (recently reviewed by Thamsborg and colleagues [42]). In order to address this question, we compared individual *Strongyloides* isolated at the same time and location from humans and dogs, which, to our knowledge, had never been done. In our study area, rural communities in Northern Cambodia, people share their premises closely with their dogs, and the prevalence of strongyloidiasis is high [3]. We compared the sequences of the nuclear *SSU* HVR I and HVR IV and, for a selected subset of worms, the mitochondrial *cox*1 gene and whole genome sequences. Further, we characterized reproduction in the free-living generations of wild and laboratory isolates of *S*. *stercoralis*. Our results show that dogs carry *S*. *stercoralis* genetically indistinguishable from the ones in humans in addition to a dog specific population. Further, we demonstrate that reproduction in the free-living generation of both wild and laboratory isolates of *S*. *stercoralis* is sexual and not pseudogamic. Overall, our observations strongly support the hypothesis that dogs are a potential source for human *S*. *stercoralis* infection and suggest that in order to reduce the exposure of people to infective *Strongyloides* larvae, dogs should be treated along with their owners in settings where people are exposed to dog excrement.

## Materials and methods

### Study area

Fecal samples were collected from humans and dogs of the same households in the villages Anlong Svay (AS) and Chom Long (CL) in May 2013 and in Damnak Chin (DC) and Kampot (KP) in June 2016. All villages are in the Rovieng District (13°21′N 105°07′E) in Preah Vihear province in Northern Cambodia.

### Stool sample collection and *S*. *stercoralis* isolation

Human stool samples were collected and *S*. *stercoralis* larvae were isolated according to established methods [47]. In brief, stool samples were collected for two consecutive days from each member of the household who agreed to participate in this study. In May 2013 all the fecal samples collected from humans were analyzed within 3 hours after collection using Baermann and Kato-Katz methods. The sediments of positive Baermann funnels were preserved and transported to our laboratory in Tübingen in 70% ethanol at ambient temperature. In June 2016 the fecal samples were mixed with an approximately equal volume of sawdust, moisturized and cultured at ambient temperature for 24–48 hours and analyzed using the Baermann method. From positive Baermann funnels a portion of the worms were transferred individually into 10 μl of water or, for those intended for whole genome sequencing, 10 μl of Tissue and Cell Lysis Solution (component of the MasterPure DNA Purification Kit, Epicenter MC85201) and the remaining worms were preserved as batches in 70% ethanol. While the work was ongoing the samples were stored in the hotel freezer. For transport to our laboratory the samples were refrigerated using wet ice but not frozen. In the majority of cases, worms from the 2016 sample come from those that had been picked individually whilst alive into water or Tissue and Cell Lysis Solution. If any ethanol preserved specimen from 2016 was used, this is explicitly stated.

Fecal samples were also collected from dogs found in the proximity of *S*. *stercoralis* positive households. The samples were taken directly from the rectum of the animals with the help of the owners and the field assistants. The samples were further processed like the human samples except that for some samples, 3 g of feces were placed on NGM agar plates [48] and incubated for 24–48 hours at ambient temperature and emerging *S*. *stercoralis* were picked directly from the plates instead of setting up saw dust cultures followed by baermanization.

### Single worm DNA preparation for PCR and whole genome sequencing

For ethanol fixed samples, single worms were picked and washed twice with Phosphate-buffered saline (PBS) and then incubated in 20 μl 1X lysis buffer (20 mM Tris-HCl pH 8. 3, 100 mM KCl, 5 mM MgCl2, 0.9% NP-40, 0.9% Tween 20, 0.02% Gelatine, 240 μg/ml Proteinase K) at 65°C for 2h, followed by incubation at 95°C for 15 min. 2 μl (for *SSU*) or 4 μl (or single copy loci) of this lysate were used as template for PCR amplification.

For worms stored in 10 μl water, 10 μl 2x lysis buffer were added, after which the samples were treated as described above. For samples preserved in Tissue and Cell Lysis Solution, single worm DNA was prepared using the MasterPure DNA Purification Kit (Epicenter MC85201) according to the manufacturer’s protocol, and the DNA was stored frozen in 10 μl of TE buffer. 1 μl was used for *SSU* amplification, and the reminder for sequencing library construction.

### PCR for *SSU*, *cox*1 and single copy locus genotyping

**Fig 2:**
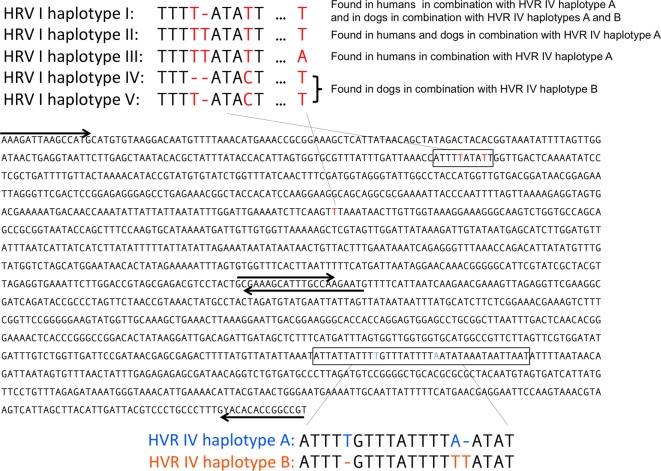
The different *SSU* HVR variants found. The sequence of the portion of the *SSU* amplified for genotyping of the two HVRs. Arrows indicate the positions of the primers used for amplification, from top to bottom SSU18A, 18SP4F, SSU26R, 18SPCR (c.f. Table 1). Note that forward primers are above the sequence while reverse primers are below the sequence. HVR I and HVR IV as defined by Hasegawa and colleagues [35] are boxed. For sequencing results for both *SSU* HVR and *cox*1 for each individual worm see S1 Table.

PCR reactions were done in a total volume of 25 μl (up to 25 μl nuclease-free water, 2.5 μl 10X ThermoPol Reaction buffer (New England BioLabs), 0.5 μl dNTP’s (2mM each), 0.5 μl 10 mM forward primer (Table 1, Fig 2), 0.5 μl 10 mM reverse primer (Table 1, Fig 2), 0.3 μl Taq DNA polymerase (New England BioLabs), 1 μl to 4 μl template as specified above). Thermocycling program: 94 °C for 2 min, followed by 35 cycles of denaturing (94 °C for 30 sec), annealing (temperature given in Table 1 for 15 sec), extension (72 °C for time given in Table 1), and a post amplification final extension (72°C for 10 min) and cooling to 4 °C.

**Table 1:** Primers and PCR conditions.

	Primer		Sequence	Ann	Ext	Prod
*SSU* HVR I	Fw	SSU18A^a^	5'-AAAGATTAAGCCATGCATG-3'	52°C	90''	863 bp
	Rev	SSU26R^a^	5'-CATTCTTGGCAAATGCTTTCG-3'			
	Seq	SSU9R	5'-AGCTGGAATTACCGCGG-3'			
*SSU* HVR IV	Fw	18SP4F^b^	5'-GCGAAAGCATTTGCCAA-3'	57°C	90''	712 bp
	Rev	18SPCR^b^	5'-ACGGGCGGTGTGTRC-3'			
*cox*1	Fw	TJ5207	5'-TTTGATTGTTACCTGCTTCTATTTT-3'	50°C	90''	650 bp
	Rev	TJ5208	5'-TTTTACACCAGTAGGAACAGCAA-3'			
*ytP274*	Fw	TJ6026	5'-CAGGACCACCTGGACAAGTT-3'	54°C	90''	543 bp
	Rev	TJ6027	5'-CTTTCCATCCTGATGCCACT-3'			
*ytP289*	Fw	ZS6420	5'-TGAAACAGGAAAACACATCTACTGA-3'	49°C	90''	765 bp
	Rev	ZS6421	5'-AGTGTTCAAGATATTCACGCAG-3'			
*ytP289* nested	Fw	ZS6472	5'-AAATGGTTCAAGTTTGGGAC-3'	49°C	60''	431 bp
	Rev	ZS6473	5'-TGACATACCATTAGCTTCACCA-3'			
*ytP290*	Fw	ZS6490	5'-TGCTGCCTCAACAATGTACA-3'	49°C	60''	431 bp
	Rev	ZS6491	5'-TTATAGGCATCTAAAAGGCTTT-3'			
*ytP290* nested	Fw	ZS6448	5'-GCTGTACAGGATGCTTTGGA-3'	49°C	60''	203 bp
	Rev	ZS6449	5'-TGTGCGATACATAATTTTCTGATGAA-3'			

^a^Taken from [10];

^**b**^ taken from [35].

Rev reverse; Seq sequencing; Fw forward; Ann annealing temperature for PCR; Ext extension time for PCR; Prod PCR product length.

### Sequencing of PCR products and sequence analysis

1 μl of the PCR reaction and either one of the PCR primers or, in the case of *SSU* HVR I, a designated sequencing primer were used in sequencing reactions using the BigDye Terminator v3.1 Cycle Sequencing Kit (Applied Biosystems,) according to the manufacture’s protocol. The reactions were submitted to the in-house sequencing facility at the Max Planck Institute for Developmental Biology at Tübingen for electrophoresis and base calling. Sequences were analyzed with SeqMan Pro version 12 (Lasergene package; DNAStar, Inc., Madison, WI USA). Chromatograms were visually inspected to detect ambiguous signals indicating mixed sequences (heterozygous worms). For comparison with published sequences, we used BLAST against the NCBI nucleotide database (http://blast.ncbi.nlm.nih.gov/Blast.cgi). For the *SSU*, we used the GenBank entry AF279916 as reference sequence. All position numbers refer to this entry. For *S*. *stercoralis cox*1, we used the sequence LC050212 as reference. 552 base pairs, of which 63 were polymorphic, were considered (for the full sequences of the different haplotypes see S1 Text). For *ytPxxx* markers, position 1 is the first base of the (non-nested) forward primer (for the sequences of the markers see S1 Text).

**Fig 3:**
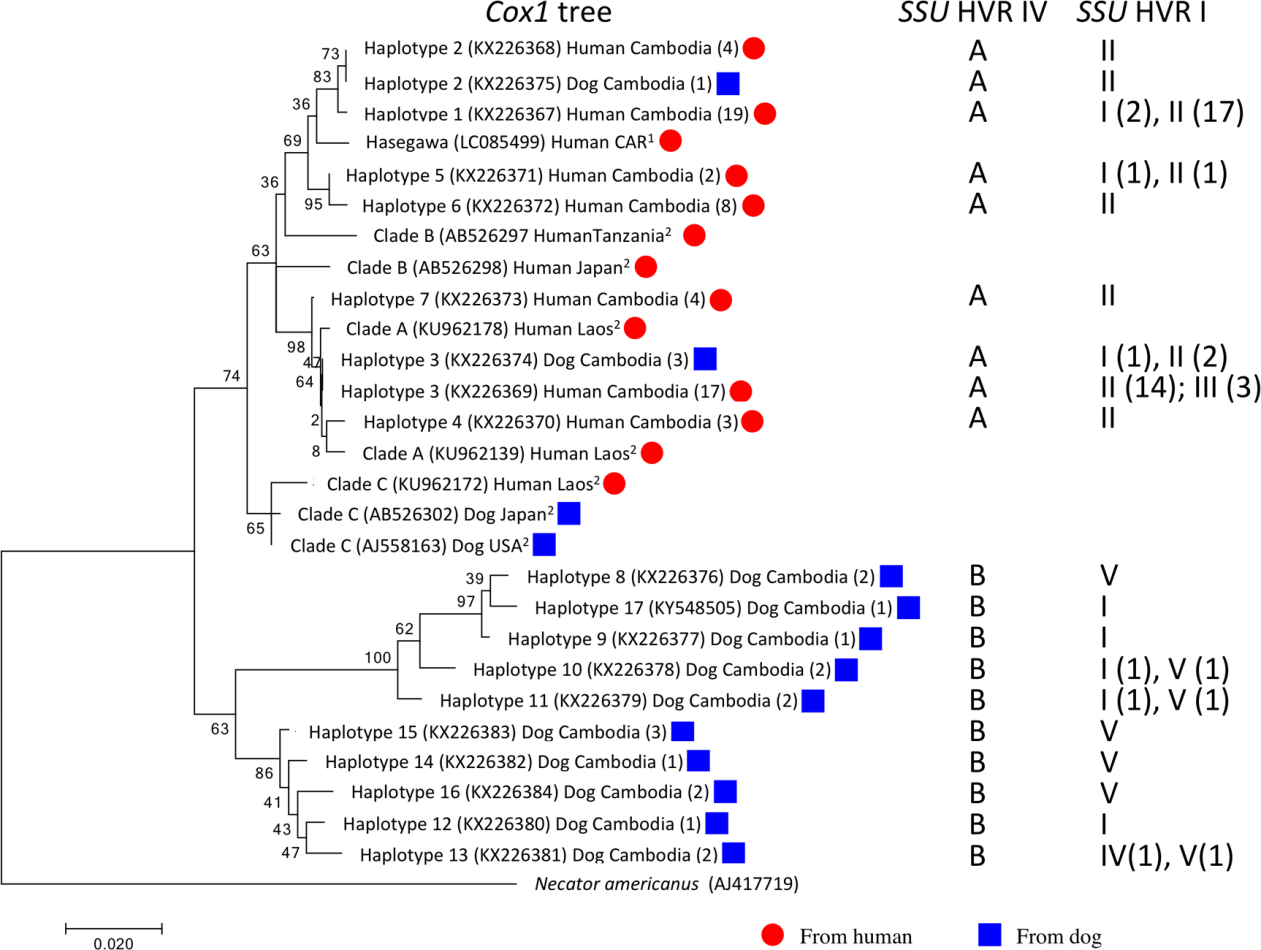
Gene tree of the mitochondrial gene *cox*1. Maximum likelihood tree of the 17 different *cox*1 sequences we found and representative previously published sequences. The numbers are bootstrap values based on 1000 bootstraps. For haplotypes isolated in this study the labels have the following format: Haplotype number (accession number) host country (number of individuals this haplotype was found in). ^1^For sequences previously published by Hasegawa and colleagues [36] the label starts with Hasegawa. ^2^For sequences previously published by Laymanifong and colleagues [6] the label starts with the *cox*1 clade this reference assigned the particular sequence to. This is followed by: (accession number) host country (CAR = Central African Republic). The hosts are also highlighted by red circles (human) and blue squares (dog). Entries to the right of the tree indicate for each *cox*1 haplotype the *SSU* haplotypes it was found together in the same individual. If a given *cox*1 haplotype existed in the context of multiple *SSU* haplotypes, the number of worms with this particular combination is given in parentheses. Note: the *cox*1 haplotypes 2 and 3 were found in both hosts and are included twice in this tree. For the sequencing results for both *SSU* HVR and *cox*1 for each individual worm see S1 Table.

Phylogenetic analysis of the *cox*1 sequences was done using MEGA7 [49] with default settings. As an outgroup species we used *Necator americanus* (AJ417719). The Maximum Likelihood tree is shown in Fig 3. For comparison we also reconstructed Maximum Parsimony and Neighbor Joining trees, which resulted in the same tree topology as far as well-supported nodes are concerned.

### Whole genome sequencing and analysis

DNA of 23 single free-living males *S*. *stercoralis* from different hosts (11 males from 9 different humans and 12 males from 10 different dogs) was prepared as described above. Whole-genome sequencing libraries were prepared with Clontech Low Input Library Prep Kits (Takara Bio, USA) following the manufacturer's protocol. Samples were submitted to in-house sequencing on an Illumina HiSeq 3000 instrument (150 bp paired-end). An approach similar to a previous *S*. *stercoralis* population study [46] was used to analyze the whole genome data. In brief, raw reads were trimmed with skewer [50] (version 0.1.123; -q 30 -Q30 -l 60). Trimmed reads were mapped to the *S*. *stercoralis* reference genome (GCA_000947215.1) and the small subunit ribosomal RNA (*SSU* rRNA; AF279916) using bwa mem [51] (version 0.7.12; MEM algorithm with defaults). Duplicate reads were marked with Picard tools (http://broadinstitute.github.io/picard) (version 2.2.1; MarkDuplicates with defaults). Variants were called with GATK [52] (build 2016-09-27-g026f7e8; HaplotypeCaller and GenotypeGVCFs both with defaults) after Indel realignment (GATK IndelRealigner with defaults) and freebayes [53] (version 1.0.2-6-g3ce827d, defaults). The intersection of both variant calls was imported into GNU R using SNPRelate [54] (version 1.8.0; method = "biallelic.only"). Single-nucleotide polymorphism (SNPs) were recursively removed using a sliding window approach and a Linkage Disequilibrium (LD) threshold of 0.05 and or a minimum allele frequency (MAF) of 0.05. The resulting SNP set was used to calculate the fraction of identity by state (IBS) for each pair of samples. Results were stored in a genomic identity-by-state relationship matrix and used to estimate a phylogeny with the BIONJ algorithm implemented in ape [55] (version 4.1; defaults). A second phylogeny was estimated with a reference-free approach. Trimmed short reads were used to generate a k-mer count graph with khmer (version 0.2.0; load-into-counting.py with a k-mer size of 19 otherwise defaults). k-mer counts were used to calculate pairwise distances between samples using kWIP [56]. The resulting distance matrix was imported into GNU R to estimate a phylogeny; again using the BIONJ algorithm.

### Analysis of free-living females and their progeny

Free-living females were isolated after two days of culture as described above and placed individually onto NGM plates seeded with *E*. *coli* OP50 [48]. The plates were inspected daily. Females that had produced progeny but presumably had ceased reproduction because they no longer contained embryos in their uterus were picked and prepared for genotyping as described above. One day later, larvae from these females were also isolated and processed. For 17 of these families the mothers and the progeny were picked individually and alive into 10 μl of water and processed as described above. Five more families (Females 7, 8, 14, 15, 16) were preserved in Ethanol (one tube per family) and the individuals were only separated when they were genotyped. The mothers and the progeny were genotyped at *ytP289* and *ytP290* as described above. *ytP289* contains single nucleotide polymorphisms at positions 308 (A/G), 359 (A/G), 416 (C/T) and 566 (C/T). Three different combinations (alleles) existed in our samples. Allele 1 has the combination G+A+C+C, allele 2 A+G+T+T, and allele 3 A+A+C+C. *ytP290* contains single nucleotide polymorphisms at positions 291 (A/C) and 310 (A/G). Two different combinations (alleles) existed in our samples. Allele 1 has the combination A+A and allele 2 C+G. For the full sequences see S1 Text.

### Crossing free-living stages of *S*. *stercoralis* (laboratory strain)

The UPD strain and PV001 line of *S*. *stercoralis* were maintained in immuno-suppressed dogs and cultured in fecal cultures with charcoal as previously described [41]. Free-living L4 larvae and young adults (males and virgin females) were isolated from 1-day fecal cultures at 22 °C using Baermann funnels as described [41].

Single virgin females and males were handpicked and transferred in male-female pairs onto NGM plates spotted with 30 μl of OP50 [48] and 20 μl of water from a Baermann funnel and incubated at 22 ^o^C for 24 hrs. On the next day, from pairs where the female contained developing embryos in the uterus, the males were transferred into PCR tubes containing 10 μl of lysis buffer (see above) and frozen for later use. Once the females contained no embryos any longer (after three days) they were processed like the males. After all the eggs had hatched, all the L1/L2 were transferred individually into PCR tubes as described for the parents. Single worm lysis was performed as described above for ethanol fixed specimens without the PBS washing step. The parents and eight progeny per cross were genotyped at the marker *ytP274* as described above. *ytP274* has a single SNP (T/C) at position 236. For the full sequence of *ytP274* see S1 Text.

### Ethics statements

The sampling of material in Cambodia was approved by the National Ethics Committee for Health Research (NECHR), Ministry of Health, Cambodia and the ethics committee of the cantons of Basel-Stadt and Basel-Land (EKBB), Switzerland. All participants were informed of the study procedures and provided written informed consent prior to enrolment. All data handled were strictly confidential. All individuals infected with *S*. *stercoralis* were treated with Ivermectin (single oral dose of 200 μg/kg BW). Co-infections with other intestinal helminths were treated according the Cambodian treatment guidelines. The experiments requiring culture of *S*. *stercoralis* in host animals were all done at the University of Pennsylvania with the approval of the University of Pennsylvania Institutional Animal Care and Use Committee (IACUC). The *S*. *stercoralis* UPD strain and PV001 isolate were maintained in prednisolone-treated dogs according to IACUC protocols 702342, 801905, and 802593. All IACUC protocols, as well as routine husbandry care of the animals, were conducted in strict accordance with the *Guide for the Care and Use of Laboratory Animals of the National Institutes of Health*. No human subjects were used in this part of the study.

### Accession numbers

The sequences obtained from this study are available from GenBank (accession numbers KU724124-KU724129 and KX226367-KX226384 and KY548505).

The whole genome data are available permanently from the FTP server of the Max Planck Institute for Developmental Biology (ftp://ftp.tuebingen.mpg.de/pub/PLOS_NTD_Jaleta_2017_whole_genome_data). They have also been submitted to the European Nucleotide Archive (accession number PRJEB20999).

## Results

### Prevalence of *S*. *stercoralis* in humans and dogs

In May 2013, we collected stool samples from a total of 537 persons from 128 households. Of these, 177 individuals (32.96%) living in 95 different households were positive for *S*. *stercoralis*. From positive households we obtained rectal fecal samples from a total of 88 dogs. Of these, 78 (88.63%) originating from 44 different households were positive. In June 2016, 20 of 169 (11.8%) people from 17 out of 62 households tested positive for *S*. *stercoralis*. Of the 29 dogs from 14 households tested, 22 (75.9%) living in 12 different households were positive. It should be noted that the prevalence in the two hosts cannot be compared directly because of the biased sampling of dogs.

### Multiple *SSU* HVR I haplotypes among *S*. *stercoralis*

Firstly, we sequenced the region around the HVR I of the *SSU* from individual *S*. *stercoralis* isolated from humans and dogs [10,34–36,27–33]. Overall, we found the five different haplotypes described in Fig 2. The two most dissimilar haplotypes (III and IV) show four differences (2 indels and 2 SNPs). Earlier studies [27–30,35,36] found this fragment to be largely invariable within species and two or more differences to be fairly reliable indicators of different species. Therefore, a within species variability as we observed was rather unexpected but not entirely implausible. In order to obtain additional information, we next sequenced the *SSU* HVR IV[35,36].

### Dogs carry two different populations of *S*. *stercoralis* identifiable by the *SSU* HVR IV sequence

**Fig 4:**
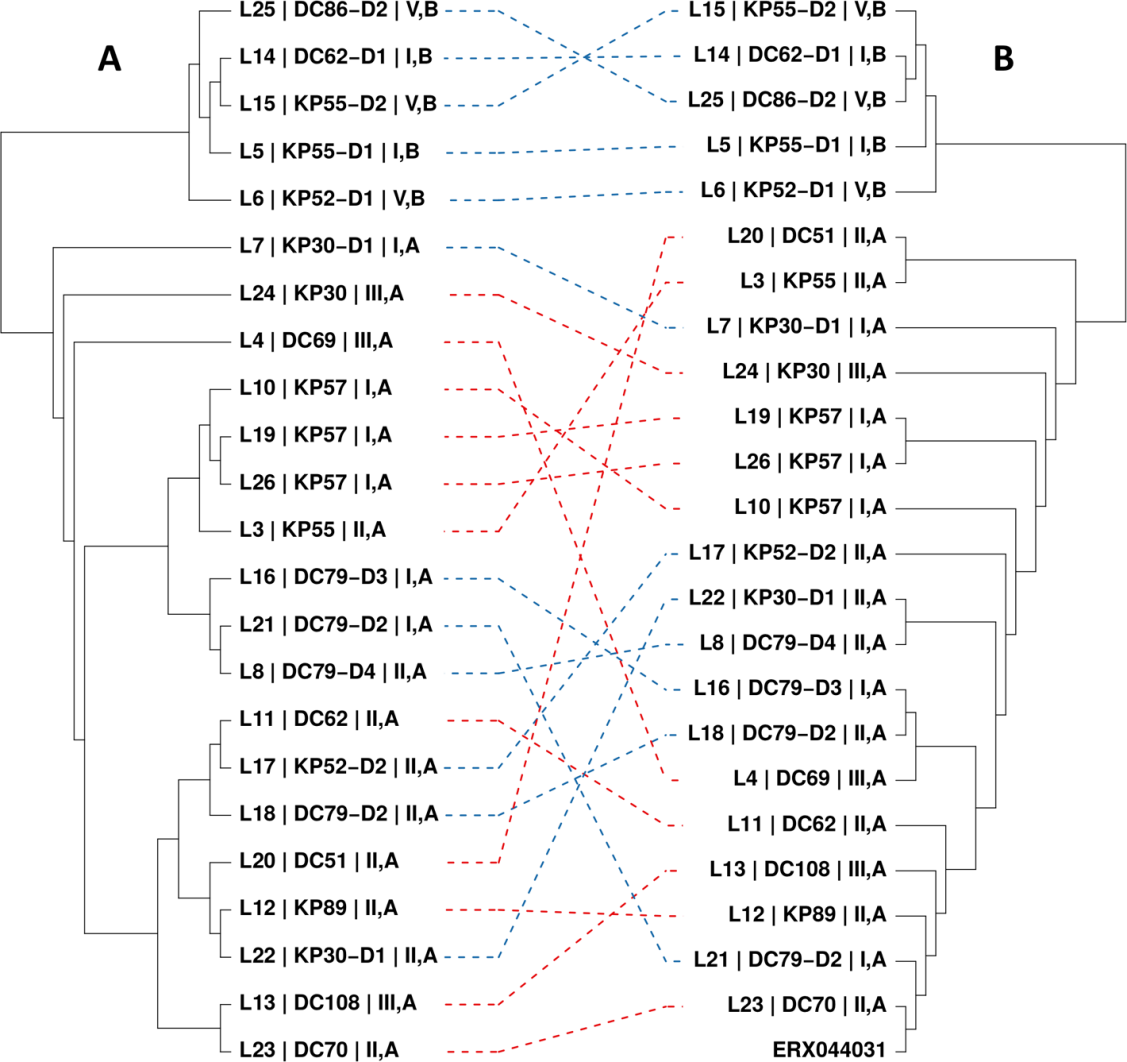
Sample relatedness analysis on whole genome data. A) Neighbor joining tree based on a genomic identity-by-state relationship matrix in cooperating 1326 SNPs (thresholds: LD = 0.05, MAF = 0.05). B) Neighbor joining tree based on pairwise similarities of the 23 individual genomes estimated using kWIP. The same samples in the two trees are connected with dotted lines colored in red for human derived worms and in blue for dog derived worms. ERX044031 indicates the reference genome short read data set [57], which has HVR I haplotype I and HVR IV haplotype A. The labels contain: [identifier of the worm] | [identifier of the host individual] | [HVR I haplotype, HVR IV haplotype], with each attribute separated by vertical lines.

Among all *Strongyloides* individuals from dogs, we found two sequence variants in the *SSU HVR IV* as defined in [35]. The two haplotypes differed at three positions (two indels, one base substitution, Fig 2). One of these variants, from now on referred to as HVR IV haplotype A, is the one previously described as the HVR IV sequence of *S*. *stercoralis* [35], and was found (Table 2) in 11 (11.5%) out of 96 dog derived worms in the 2013 sample and 39 (31%) out of 126 dog derived worms in the 2016 sample (in total 50 (22.5%) out of 222). This variant was also found in all human derived worms of both samples (in total 521 worms from a total of 85 host individuals, S1 Table). The other variant, referred to hereafter as HVR IV haplotype B, has, to our knowledge, not been described previously and was present in 85/96 (88.5%) dog derived worms in the 2013 sample, and in 87/126 (69%) dog derived worms in the 2016 sample. Notably, this haplotype was not found in any of the 521 human derived worms. If the two haplotypes indeed indicate two reproductively isolated groups, which have been separated for some time in evolution, this should also be reflected in their mitochondrial and nuclear genomes. Therefore, from the 2013 sample we sequenced a portion of the mitochondrial *cox*1 gene of 21 dog-derived worms (17 with *SSU* HVR IV haplotype B and 4 with *SSU* HVR IV haplotype A) and 57 human derived worms (all with *SSU* HVR IV haplotype A) [6,36,38]. In total we identified 17 different *cox*1 haplotypes. Seven were associated with *SSU* HVR IV haplotype A and 10 with *SSU* HVR IV haplotype B (Fig 3, S1 Table). No haplotypes were shared between worms with the two *SSU* HVR IV haplotypes. In a phylogenetic analysis, the *cox*1 haplotypes associated with the two HVR IV haplotypes were well separated from each other. From the 2016 sample we selected 23 worms for whole genome sequencing and compared them over their entire genome using two different approaches (Fig 4). Both methods robustly separated the worms with *SSU* HVR IV haplotype B form all worms with *SSU* HVR IV haplotype A. The two methods yielded different results on the exact topology of the tree within the two *SSU* HVR IV defined groups but provided no indication that the dog derived worms with *SSU* HVR IV haplotype A form a group different from the human derived worms. From this we conclude that dogs in our study area carry two populations of *S*. *stercoralis*, which are distinguishable by their *SSU* HVR IV haplotype. One population, to which the majority of the *Strongyloides* belong, was not found humans. The other population, however, is shared with humans, strongly indicating that *S*. *stercoralis* with *SSU* HVR IV haplotype A can shuttle between the two vertebrate host species.

**Table 2:** *SSU* HVR IV haplotype distribution in *S*. *stercoralis* from humans and dogs.

	From dogs			From humans		
HVR IV haplotype	A	B	% A	A	B	% A
2013 sample	11	85	11.5%	340	0	100%
2016 sample	39	87	31.0%	181	0	100%
Total	50	172	22.5%	521	0	100%

### Different HVR I haplotypes appear not to reflect genetically separated populations

When we analyzed the region around HVR I of *S*. *stercoralis* with HVR IV haplotype A, we found the same three variants (haplotypes I, II, III c.f. Fig 2) as described by Schär, Guo and colleagues [37] at various frequencies (Table 3). In combination with HVR IV haplotype B, which occurs only in dogs, we also found three variants of HVR I (haplotypes I, IV, V c.f. Fig 2). It is noteworthy that only HVR I haplotype I occurred in combination with both HVR IV haplotypes (c.f. Fig 2) [37]. If the different HVR I haplotypes reflect at least partially separated populations this should be reflected in the whole nuclear genome and the separation should also be visible in the mitochondrial genome. However, the mitochondrial *cox*1 phylogeny is not correlated with the HVR I differences (Fig 3), indicating that there is mixing of the nuclear genome between the different mitochondrial matrilinages. In addition, data from whole genome sequencing (Fig 4) are not consistent with the null hypothesis that worms with the same HVR I haplotype are more closely related to each other than to individuals with a different HVR I haplotype. The two methods lead to different tree topologies, none of which correlates with the HVR I haplotypes. Therefore this analysis provides no evidence for the existence of genetically isolated subpopulations within the worms with HVR IV haplotype A. The same is true for the worms with HVR IV haplotype B and different HVR I haplotypes. These results show that among the worms analyzed, other than *SSU* HVR IV haplotypes, not every *SSU* HVR I haplotype appears to represent its own separate genetically isolated population.

**Table 3:** *SSU* HVR I haplotypes of *S*. *stercoralis* with HVR IV haplotype A isolated from humans and dogs, 2013 and 2016.

	2013		2016	
HVR-I haplotype	From humans	From dogs	From humans	From dogs
I	28	5	5	8
II	298	6	153	31
III	14	0	23	0
Total	340	11	181	39

### *S*. *stercoralis* free-living adults reproduce sexually

As a possible explanation for the absence of hybrids, Schär, Guo and colleagues [37] proposed that *S*. *stercoralis* might not reproduce sexually, either because the free-living generation undergoes pseudogamy (sperm dependent parthenogenesis) as proposed earlier [25], or because in the study area the population propagates only through the non-sexual homogonic cycle. We showed above that the nuclear genome and the mitochondrial genomes, which are normally maternally inherited, do not evolve in parallel. This argues strongly for at least occasional sexual reproduction. Large numbers of free-living *S*. *stercoralis* of both sexes were present in our field and laboratory isolates and males were clearly required for reproduction in the free-living generation. Of a total of 480 females (96 from field isolates and 384 from a laboratory isolate) placed individually on plates before they had a chance to mate, none produced progeny. This result indicates that female autonomous reproduction by parthenogenesis or self-fertilization occurs rarely or not at all. However, the result is not an argument against pseudogamy, because, although they do not contribute genetic material to the progeny, males and their sperm are required to activate embryogenesis in this mode of reproduction. Further, although mitochondria are normally inherited only from the mother, in other nematodes male derived mitochondria occasionally fail to be degraded and are incorporated in zygotes [58,59]. This could also happen upon pseudogamic interaction between oocyte and sperm and lead to a recombination of nuclear and mitochondrial genomes. Therefore, we sought to demonstrate sexual reproduction more directly.

To this end we genotyped 22 individual mothers (all derived from humans) and several of their progeny at two single-copy loci (Table 4). These results demonstrate clearly that the progeny were not the product of clonal reproduction because we found larvae with genetic material absent from the mother but presumably derived from the male and/or larvae that did not have alleles present in the mother, indicating that the mother passed on only half of its genetic material as expected for sexual but not for clonal reproduction.

**Table 4:** Genotypes of free-living mothers and their progeny.

Female number	Host individual^a^	Informative marker and female Genotype^2^	Progeny genotypes
F1	DC51	*ytP289* 1/2	2x 1/1, 1x 2/2
F2	DC51	*ytP289* 1/2	2x 1/1, 2x 2/2
F3	DC51	*ytP289* 1/2	3x 1/1, 3x 2/2
F4	DC51	*ytP289* 1/2	1x 2/2
F5	DC51	*ytP289* 1/1	3x 1/1, 1x 1/2
F6	DC51	*ytP289* 1/2	3x 1/2, 2x 2/2
F7	DC51	*ytP289* 1/2	1x 1/1, 3x 1/2, 4x 2/2
F8	DC51	*ytP289* 1/2	2x 1/1, 3x 1/2, 2x 2/2
F9	KP57	*ytP289* 1/1	1x 1/1, 2x 1/3
F10	KP57	*ytP289* 2/3	1x 1/2, 5x 2/3, 4x 3/3
F11	KP57	*ytP289* 1/3	6x 1/2, 2x 1/3
F12	KP57	*ytP289* 2/3	1x 2/2, 1x 2/3
F13	KP57	*ytP289* 2/3	4x 1/2, 1x 1/3, 1x 2/2
F14	DC108	*ytP289* 1/2	1x 1/1, 2x 1/2
F15	DC108	*ytP289* 1/2	1x 1/1
F16	DC108	*ytP289* 1/2	2x 1/2, 3x 2/2
F17*	DC69	*ytP289* 1/2	1x 1/2, 1x 1/1
F17*	DC69	*ytP290* 1/2	3x 1/2, 2x 1/1, 2x 2/2
F18	KP31	*ytP290* 1/2	3x 1/1, 2x 2/2
F19	KP31	*ytP290* 1/2	2x 1/1, 8x 1/2, 5x 2/2
F20	KP31	*ytP290* 1/2	1x 1/1, 6x 1/2, 4x2/2
F21	KP31	*ytP290* 1/2	2x 1/1, 1x 1/2
F22	KP31	*ytP290* 1/2	3x 1/2, 1x 1/1, 2x 2/2

^a^The host individual is defined by the two-letter code for the village followed by the host individual number.

^2^*ytP289* contains single nucleotide polymorphisms at positions 308 (A/G), 359 (A/G), 416 (C/T) and 566 (C/T).

Allele 1 has the combination G+A+C+C, allele 2 A+G+T+T, and allele 3 A+A+C+C. *ytP290* contains single nucleotide polymorphisms at positions 291 (A/C) and 310 (A/G). Allele 1 has the combination A+A, allele 2 C+G.

*F17 was the only case where both markers were informative.

We sought to further solidify this in a fully controlled experiment with known males and a larger number of progeny. To this end, we set up crosses with single females and males using a laboratory isolate of *S*. *stercoralis*. The results (Table 5) were fully consistent with Mendelian inheritance with equal genetic contribution by males and females but not with clonal reproduction.

**Table 5:** Genotypes of free-living parents and their progeny at *ytP274*.

Cross	Female genotype^a^	Male genotype^a^	Progeny genotypes^a^
C1	C/T	C/T	4x C/T, 1x C/C, 3x T/T
C2	C/T	C/T	4x C/T, 2x T/T, 2x C/C
C3	C/T	C/C	4x C/C, 4x C/T
C4	T/T	C/T	4x C/T, 4x T/T
C5	T/T	C/T	4x C/T, 4x T/T
C6	C/C	C/T	3x C/C, 5x C/T
C7	C/T	C/C	4x C/C, 4x C/T
C8	C/T	T/T	3x C/T, 5xT/T
C9	T/T	C/T	4xC/T, 4x T/T
C10	C/C	C/T	4x C/C, 4xC/T
C11	C/T	T/T	4x C/T, 4x T/T
C12	C/T	C/T	4x C/C, 3x C/T, 1x T/T
C13	C/T	C/T	5x C/T, 1x C/C, 2x T/T
C14	C/T	C/T	4x C/T, 1x C/C, 3x T/T
C15	C/T	C/C	5x C/C, 3x C/T
C16	C/T	C/T	3x C/T, 2x C/C, 3x T/T
C17	C/C	C/T	3x C/T, 5x C/C
C18	T/T	C/T	6x C/T, 2x T/T

^a^The marker *ytP274* has a single nucleotide polymorphism (T/C) at position 236.

### Are there hybrids between *SSU* haplotypes?

Given the results above, one would expect to observe animals that are hybrids between the different *SSU* HVR I haplotypes. However, like in an earlier study [37], we failed to find any hybrids among the 436 larvae from 97 different host individuals (68 humans 29 dogs) isolated in 2013. The *SSU* locus is on the X chromosome in *S*. *stercoralis* (information extracted from a previously published dataset [57]). Therefore, hybrid males could not be detected because they only contain one X chromosome. However, for [37] and in our 2013 sampling, young larvae that were the progeny of parasitic worms were analyzed. The sex of these larvae was unknown but given that the field isolates produced both sexes, it is very likely that a substantial number of females were among them. In 2016 we tested adult worms and used predominantly males because, unlike females, they may not contain genetic material from other individuals (sperm, embryos). Only 30 of the 307 worms from 2016 listed in Table 2 were pre-reproductive females. None of them was a hybrid.

However, of the gravid females described in Table 4 one (Female 17) and one of its progeny showed a mixture of HVR I haplotypes II and III. While the mixed signal in the female might have been caused by sperm and embryos derived from a male with a different haplotype than the mother, the larva was most likely a true hybrid. This prompted us to test fully mature females that had been isolated from cultures, after they had a chance to mate, preserved in ethanol and separated by host individuals. We analyzed 9 to 23 worms from each of six host individuals (four humans, two dogs), from which we had already isolated *S*. *stercoralis* of different HVR I haplotypes. In five of the cases we found worms with mixed haplotypes, along with individuals with only one *SSU* haplotype (Table 6). Two observations are noteworthy. Firstly, both dogs had worms with HVR IV haplotypes A and B. In total, 11 out of 21 worms had mixed HVR I haplotypes but only a single one showed a mixture between the two HVR IV haplotypes, which might indicate that males and females with different HVR IV haplotypes tend to avoid each other. Secondly, from host individual KP57 (human), 9 of 14 worms had mixed haplotypes. From the same host individual we had also genotyped 12 pre-reproductive females, none of which was a hybrid. This finding suggests that the females genotyped in this experiment were not true hybrids but contained sperm and developing embryos derived from males of the other haplotype detected.

**Table 6:** *SSU* haplotypes found in individual gravid females.

Host (species)	Non-hybrid genotypes HVR I + HVR IV (number of individuals)	Mixed genotypes HVR I + HVR IV (number of individuals)	Mixed/Total
DC44 (Human)	II+A (12)	-	0/12
DC69 (Human)	II+A (6), III+A (3)	II/III+A (14)	14/23
KP30 (Human)	II+A (3)	II/III+A (8)	8/11
KP57 (Human)^a^	II+A (5)	I/II+A (9)	9/14
DC79D2 (Dog)	II+A (4), V+B (1)	I/II+A (1), V/IV+B (3)	4/9
KP52D2 (Dog)	II+A (4), IV+B (1)	V/IV+B (6), V/II+A/B (1)	7/12

^a^from this host individual we also genotyped 12 pre-reproductive females and 19 males.

All females and 14 males were of haplotype II+A, 5 males were I+A.

## Discussion

In rural communities in Cambodia, many people share their premises with domestic animals and the general hygenic, water and sanitation infrastructures are precarious [60,61]. Therefore, the conditions appear very favorable for human to animal and animal to human transmission of STH including *S*. *stercoralis* [3]. In order to find evidence for or against zoonotic transmission of *S*. *stercoralis* under such circumstances, we isolated large numbers of *S*. *stercoralis* from humans and dogs at the same time and in the same households and analyzed individual worms using molecular genetic markers. To our knowledge there had been no such study of *S*. *stercoralis* of comparable scale undertaken anywhere. It should be noted that our experimental strategy aimed to sample individuals with a large potential for transfer of *Strongyloides* spp. between the two hosts (e.g., only dogs found close to households with positive people were sampled). Therefore our study was not designed to yield accurate estimates of haplotype frequencies in the entire population. We also point out that we did not directly demonstrate transmission from dogs to humans and therefore cannot exclude that the transmission is mostly or exclusively from human to dog. Nevertheless, our results strongly suggest that there is a considerable risk for dog to human transmission. This would not be in agreement with conclusions by Takano and colleagues [62] who found that humans in households with *Strongyloides*-infected dogs were not more likely to be parasitized by *S*. *stercoralis* than those with parasite free dogs and concluded that natural transmission does not occur between humans and dogs. However, this study was conducted in Japan, in areas with presumably much better sanitary conditions than in the Cambodian villages where the present study was conducted. Consequently, only five *Strongyloides* positive dogs were found and none of their owners was infected. Likewise, a study conducted in Southern China, in a setting probably more comparable to our study area [47], did not identify the presence of animals as a statistically significant risk factor for human strongyloidiasis. However, this conclusion was based on only 21 infected individuals (11.7% of the tested), and no details about the exposure to dogs are given. In rural settings dogs are usually semi-domesticated and roam freely such that the risk of exposure to contamination by canine feces among people who do not own a dog themselves might be approximately equal to the risk among people who do. Therefore, the lack of statistical significance cannot be taken as evidence against zoonotic transmission. Interestingly, a later study in a similar setting [63] revealed that anthelmintic treatment of people alone was not sufficient to significantly reduce the prevalence of *S*. *stercoralis*.

Overall, the present findings strongly suggest that dogs must be seriously considered as sources for human strongyloidiasis. Whether dogs are the only non-human carrier of concern or if other animals also have the same potential remains to be determined. However, our results also show that at least in our study area, the majority of the *Strongyloides* present in dogs is of a genotype that we never found humans. Therefore, the number of *Strongyloides* spp. detected in dogs by coproscopic diagnosis might be an inaccurate index of the risk of exposure to *S*. *stercoralis* that dogs pose to humans.

The 18S rDNA HVR IV appears to be diagnostic for the two separate *Strongyloides* populations in dogs. This agrees with the findings of Hasegawa and colleagues [35] who found this region to be invariable within *S*. *stercoralis*. Both, the mitochondrial *cox*1 and whole genome sequence analyses confirmed that the two HVR IV variants represent separate phylogenetic groups. Therefore, our results support the proposal by Brumpt and Augustine [43,44] of a separate species, *Strongyloides canis*.

Consistent with [37] we found three different 18S rDNA HVR-I genotypes in human derived *S*. *stercoralis*. Worms of different haplotypes sometimes co-existed in the same host individual. In comparison with other nematodes [27–33] it is unusual that such differences in this region of the *SSU* occur within one species. However, species status can never be inferred from sequence information alone. Nevertheless, in those examples in nematodes where the same fragment around the HVR I was used and more rigorous criteria for species separation (e.g. mating experiments) could be applied, a sequence difference of more than one position was a safe indicator of a distinct species [28–33]**.** Most of the time, this region appears completely invariable within a species and there are several examples where even separate species do not differ in their HVR I. However, our comparative analyses of the mitochondrial *cox*1 locus and of whole genome data suggest that in *S*. *stercoralis*, different HVR I genotypes do not indicate separate species, but rather that in *S*. *stercoralis* the HVR I is more variable than in other nematodes. It should be noted that all the well-studied cases mentioned above involve obligate sexual nematode species, some of which are capable of self-fertilization. Asexual reproduction through the homogonic cycle, which may be a frequent mode of reproduction in *S*. *stercoralis*, might contribute to a higher variability within the species. Nevertheless, although we show that the different HVR I genotypes are not diagnostic for different species, our results do not exclude the existence of cryptic species among *S*. *stercoralis*. It is striking that, like Schar et al. [37], we failed to detect hybrids between different HVR I haplotypes among all worms that were the progeny of parasitic mothers and that were definitely unmated. However, in mature free-living females and in their progeny, we frequently found mixed signals. While, for reasons described above, we doubt that these females were true hybrids, their offspring presumably were. We think that this indicates that adults of different HVR I haplotype do mate, at least in laboratory fecal cultures, and that at least some of the progeny develop to larval stages. We can only speculate about why such hybrids are not found in the progeny of the parasitic generation. It might be that the hybrids, or even the progeny of the free-living generation in general, are sub-viable and only rarely develop into successful fertile parasitic females. In this case genetic mixing between subpopulations would occur only rarely. Over long periods of time, even rare exchange might be significant and cause enough mixing of the genomes that we were not able to detect genetic differentiation between subpopulations. Alternatively, in the *S*. *stercoralis* populations in our study area there might be very high inbreeding (brother sister mating) under natural conditions. This would lead to a very high degree of homozygosity in the population. Both scenarios described above would lead to a very low number of *SSU* haplotype heterozygotes in the population, and we may have simply missed the rare hybrids. In order to address this, controlled crosses and experimental infections are required. In the context of this study we had no opportunity to do such experiments because, for logistic and for legal reasons, we were not able to bring live worms into our laboratory.

Our results demonstrate, however, that such crossing experiments are possible. We demonstrate that in contrast to earlier claims [25], sexual reproduction in the free-living generation of *S*. *stercoralis* does occur and is likely the predominant, if not the only the mode of reproduction in this generation. With this, the number of species of *Strongyloides* for which genetic analyses demonstrated that the reproduction in the free-living generation is sexual rises to four out of four tested (*S*. *ratti* [23,24], *S*. *papillosus* [22], *S*. *vituli* [64], *S*. *stecoralis*, this study). Sexual reproduction by *S*. *stercoralis* has also been confirmed recently by experimental crossing of free-living male and female worms harboring discrete reporter transgenes[65]. Although these findings do not exclude that asexual species or strains might exist, even within what is currently referred to as *S*. *stercoralis*, they do suggest strongly that sexual reproduction rather than pseudogamy is the prevailing mode of reproduction in free-living *Strongyloides* spp.

## Conclusions and outlook

Our results provide a compelling solution for the long-standing controversy about whether the *Strongyloides* sp. of dogs is identical to the *S*. *stercoralis* of humans or not. In fact, both scenarios appear to be true. Dogs, at least in our study area, host two different populations. These either represent separate species or well-separated sub-species of *Strongyloides* spp, and only one of them is shared with humans. It remains to be determined if the different types of *Strongyloides* we observed in humans and dogs also occur in other regions of the world. Among *S*. *stercoralis* in humans there is variability in the rDNA sequence. While we did not find further genomic evidence supporting multiple genetically separate populations in humans the absence of hybrids between the different *SSU* HVR I haplotypes is striking. It will be most interesting to ascertain whether different *SSU* HVR I types indeed interbreed and, even more importantly, if they might be associated with different clinical outcomes. Therefore, we suggest using molecular diagnostics for *Strongyloides* spp. wherever possible. In order to generate comparable data, we propose following the lead of Hasegawa and colleagues [35,38], and using the *SSU* HVRs I and IV and the mitochondrial *cox*1 locus as primary markers as was done in this study. With respect to strongyloidiasis control and prevention, this study suggests that dogs should be seriously considered as a source for human *S*. *stercoralis* infection at least in settings similar to our study area. Prevention of human contact with dog feces and of dog contact with human excrement as well as anthelmintic treatment of dogs are likely to reduce the exposure of humans to infective *S*. *stercoralis* larvae.

## Supporting information

S1 Text Sequences of the *cox1* haplotypes and the molecular markers. The sequences of the mitochondrial *cox1* haplotypes and the sequences of the nuclear molecular markers are given. GenBank accession numbers are in (KX226367-KX226384). For the nuclear markers the polymorphic positions are indicated by red boxes.
(DOCX)

S1 Table *SSU* HVR I and IV and *cox 1* haplotypes of all individuals analyzed. Sheet 1: worms collected 2016 from humans. Sheet 2: worms collected 2016 from dogs. Sheet 3: worms collected 2013 from humans. Sheet 4: worms collected 2013 from dogs.
(XLSX)
