# Supplementary material for: Different but overlapping populations of Strongyloides stercoralis in dogs and humans—Dogs as a possible source for zoonotic strongyloidiasis
Source: PLoS Negl Trop Dis. 2017 Aug 9;11(8):e0005752. doi: 10.1371/journal.pntd.0005752 (PMC5565190; doi:10.1371/journal.pntd.0005752)
Supplement: S1 Text — The sequences of the mitochondrial cox1 haplotypes and the sequences of the nuclear molecular markers are given. GenBank accession numbers are in (KX226367-KX226384). For the nuclear markers the polymorphic positions are indicated by red boxes. (DOCX) [file pntd.0005752.s001.docx]

**S1 Text**

***cox*1 haplotypes**

>Haplotype 1 (KX226367)

ACTAGTTGAACTATTTACCCTCCTTTATCTACTTCAGGTCATCCTGGTTCTAGTGTTGATTTGGCTATTTTCAGTTTACATCTTTCTGGTATTAGTTCTATTTTAGGTGGTATTAATTTTATGTGTACTGTTAAAAATTTACGTTCTAGTTCTGTTTCTCTTAATAATATGAGTTTATTTATTTGGACTATTTTTGTTACTGTTTTTTTATTGGTTTTGTCTTTACCTGTTTTAGCTGGTGCTATTACTATGTTGTTAATTGATCGTAATTTTAATGGTTCTTTTTTTGATCCTAGTTTTGGTGGTAATCCTTTGATTTATCAGCATTTGTTTTGGTTTTTTGGTCATCCGGAAGTTTATATTTTAATTTTACCTGCTTTCGGTATTATTAGTCAATGTACTTTATATTTGACTGGTAAAAAAGAAGTTTTTGGTTATTTGGGTATGGTTTATGCTATTTTAAGTATTGGTTTAATTGGTTGTGTAGTTTGAGCTCATCACATGTATACTGTTGGTATGGATTTTGATTCTCGTGCTTATTTTACTGCTGCT

>Haplotype 2* (KX226368)

ACTAGTTGAACTATTTACCCTCCTTTATCTACTTCAGGTCACCCTGGTTCTAGTGTTGATTTGGCTATTTTCAGTTTACATCTTTCTGGTATTAGTTCTATTTTAGGTGGTATTAATTTTATGTGTACTGTTAAAAATTTACGTTCTAGTTCTGTTTCTCTTAATAATATGAGTTTATTTATTTGGACTATTTTTGTTACTGTTTTTTTATTGGTTTTGTCTTTACCTGTTTTAGCTGGTGCTATTACTATGTTGTTAATTGATCGTAATTTTAATGGTTCTTTTTTTGATCCTAGTTTTGGTGGTAATCCTTTGATTTATCAGCATTTGTTTTGGTTTTTTGGTCATCCGGAAGTTTATATTTTAATTTTACCTGCTTTCGGTATTATTAGTCAATGTACTTTATATTTGACTGGTAAAAAAGAAGTTTTTGGTTATTTGGGTATGGTTTATGCTATTTTAAGTATTGGTTTAATTGGTTGTGTAGTTTGAGCTCATCACATGTATACTGTTGGTATGGATTTTGATTCTCGTGCTTATTTTACTGCTGCT

>Haplotype 2* (KX226375)

ACTAGTTGAACTATTTACCCTCCTTTATCTACTTCAGGTCACCCTGGTTCTAGTGTTGATTTGGCTATTTTCAGTTTACATCTTTCTGGTATTAGTTCTATTTTAGGTGGTATTAATTTTATGTGTACTGTTAAAAATTTACGTTCTAGTTCTGTTTCTCTTAATAATATGAGTTTATTTATTTGGACTATTTTTGTTACTGTTTTTTTATTGGTTTTGTCTTTACCTGTTTTAGCTGGTGCTATTACTATGTTGTTAATTGATCGTAATTTTAATGGTTCTTTTTTTGATCCTAGTTTTGGTGGTAATCCTTTGATTTATCAGCATTTGTTTTGGTTTTTTGGTCATCCGGAAGTTTATATTTTAATTTTACCTGCTTTCGGTATTATTAGTCAATGTACTTTATATTTGACTGGTAAAAAAGAAGTTTTTGGTTATTTGGGTATGGTTTATGCTATTTTAAGTATTGGTTTAATTGGTTGTGTAGTTTGAGCTCATCACATGTATACTGTTGGTATGGATTTTGATTCTCGTGCTTATTTTACTGCTGCT

>Haplotype 3* (KX226374)

ACTAGTTGAACTATTTATCCCCCTTTATCTACTTCAGGACATCCTGGTTCTAGTGTTGATTTGGCTATTTTTAGTTTACATCTTTCTGGTATTAGTTCTATTTTGGGTGGTATTAATTTTATGTGTACTGTTAAAAATTTACGTTCTAGTTCTGTTTCTCTCAATAATATGAGTTTATTTATTTGAACTATTTTTGTTACTGTTTTTTTATTGGTTTTGTCTTTACCTGTTTTAGCTGGTGCTATTACTATGTTGTTAATTGATCGTAATTTTAATGGTTCTTTTTTTGATCCTAGTTTTGGTGGTAATCCTTTGATTTATCAGCATTTGTTTTGGTTTTTTGGTCATCCAGAAGTTTATATTTTAATTTTACCTGCTTTCGGTATTATTAGTCAATGTACTTTGTATTTGACTGGTAAAAAAGAAGTTTTTGGTTATTTGGGTATGGTTTATGCTATTTTGAGTATTGGTTTAATTGGTTGTGTAGTTTGAGCTCATCACATGTATACTGTTGGTATAGATTTTGATTCTCGTGCTTATTTTACTGCTGCT

>Haplotype 3* (KX226369)

ACTAGTTGAACTATTTATCCCCCTTTATCTACTTCAGGACATCCTGGTTCTAGTGTTGATTTGGCTATTTTTAGTTTACATCTTTCTGGTATTAGTTCTATTTTGGGTGGTATTAATTTTATGTGTACTGTTAAAAATTTACGTTCTAGTTCTGTTTCTCTCAATAATATGAGTTTATTTATTTGAACTATTTTTGTTACTGTTTTTTTATTGGTTTTGTCTTTACCTGTTTTAGCTGGTGCTATTACTATGTTGTTAATTGATCGTAATTTTAATGGTTCTTTTTTTGATCCTAGTTTTGGTGGTAATCCTTTGATTTATCAGCATTTGTTTTGGTTTTTTGGTCATCCAGAAGTTTATATTTTAATTTTACCTGCTTTCGGTATTATTAGTCAATGTACTTTGTATTTGACTGGTAAAAAAGAAGTTTTTGGTTATTTGGGTATGGTTTATGCTATTTTGAGTATTGGTTTAATTGGTTGTGTAGTTTGAGCTCATCACATGTATACTGTTGGTATAGATTTTGATTCTCGTGCTTATTTTACTGCTGCT

>Haplotype 4 (KX226370)

ACTAGTTGAACTATTTATCCCCCTTTATCTACTTCAGGACATCCTGGTTCTAGTGTTGATTTGGCTATTTTTAGTTTACATCTTTCTGGTATTAGTTCTATTTTGGGTGGTATTAATTTTATGTGTACTGTTAAAAATTTACGTTCTAGTTCTGTTTCTCTCAATAATATAAGTTTGTTTATTTGAACTATTTTTGTTACTGTTTTTTTATTGGTTTTGTCTTTACCTGTTTTAGCTGGTGCTATTACTATGTTGTTAATTGATCGTAATTTTAATGGTTCTTTTTTTGATCCTAGTTTTGGTGGTAATCCTTTGATTTATCAGCATTTGTTTTGGTTTTTTGGTCATCCAGAAGTTTATATTTTAATTTTACCTGCTTTCGGTATTATTAGTCAATGTACTTTGTATTTGACTGGTAAAAAAGAAGTTTTTGGTTATTTGGGTATGGTTTATGCTATTTTGAGTATTGGTTTAATTGGTTGTGTAGTTTGAGCTCATCACATGTATACTGTTGGTATAGATTTTGATTCTCGTGCTTATTTTACTGCTGCT

>Haplotype 5 (KX226371)

ACTAGTTGAACTATTTATCCTCCTTTATCTACTTCAGGTCACCCTGGTTCTAGTGTTGATTTGGCTATTTTCAGTTTACATCTTTCTGGTATTAGTTCTATTTTAGGTGGTATTAATTTTATGTGTACTGTTAAAAATTTACGTTCTAGTTCTGTTTCTCTTAATAATATGAGTTTATTTATTTGGACTATTTTTGTTACTGTTTTTTTATTGGTTTTGTCTTTACCTGTTTTAGCTGGTGCTATTACTATGTTGTTAATTGATCGTAATTTTAATGGTTCTTTTTTTGATCCTAGTTTTGGTGGTAATCCTTTGATTTATCAGCATTTGTTTTGGTTTTTTGGTCATCCAGAAGTTTATATTTTAATTTTACCTGCTTTCGGTATTATTAGTCAATGTACTTTATATTTGACTGGTAAAAAAGAAGTTTTTGGTTATTTGGGTATGGTTTATGCTATTTTAAGTATTGGTTTGATTGGTTGTGTAGTTTGGGCTCATCACATGTATACTGTTGGTATAGATTTTGATTCTCGTGCTTATTTTACTGCTGCT

>Haplotype 6 (KX226372)

ACTAGTTGAACTATTTATCCTCCTTTGTCTACTTCAGGTCACCCTGGTTCTAGTGTTGATTTGGCTATTTTCAGTTTACATCTTTCTGGTATTAGTTCTATTTTAGGTGGTATTAATTTTATGTGTACTGTTAAAAATTTACGTTCTAGTTCTGTTTCTCTTAATAATATGAGTTTATTTATTTGGACTATTTTTGTTACTGTTTTTTTATTGGTTTTGTCTTTACCTGTTTTAGCTGGTGCTATTACTATGTTGTTAATTGATCGCAATTTTAATGGTTCTTTTTTTGATCCTAGTTTTGGTGGTAATCCTTTGATTTATCAGCATTTGTTTTGGTTTTTTGGTCATCCAGAAGTTTATATTTTAATTTTACCTGCTTTCGGTATTATTAGTCAATGTACTTTATATTTGACTGGTAAAAAAGAAGTTTTTGGTTATTTGGGTATGGTTTATGCTATTTTAAGTATTGGTTTGATTGGTTGTGTAGTTTGGGCTCATCACATGTATACTGTTGGTATAGATTTTGATTCTCGTGCTTATTTTACTGCTGCT

>Haplotype 7 (KX226373)

ACTAGTTGAACTATTTATCCCCCTTTATCTACTTCAGGACATCCTGGTTCTAGTGTTGATTTGGCTATTTTTAGTTTACATCTTTCTGGTATTAGTTCTATTTTAGGTGGTATTAATTTTATGTGTACTGTTAAAAATTTACGTTCTAGTTCTGTTTCTCTCAATAATATGAGTTTATTTATTTGAACTATTTTTGTTACTGTTTTTTTATTGGTTTTGTCTTTACCTGTTTTAGCTGGTGCTATTACTATGTTGTTAATTGATCGTAATTTTAATGGTTCTTTTTTTGATCCTAGTTTTGGTGGTAATCCTTTGATTTATCAGCATTTGTTTTGGTTTTTTGGTCATCCAGAAGTTTATATTTTAATTTTACCTGCTTTCGGTATTATTAGTCAATGTACTTTGTATTTGACTGGTAAAAAAGAAGTTTTTGGTTATTTGGGTATGGTTTATGCTATTTTGAGTATTGGTTTAATTGGTTGTGTAGTTTGAGCTCATCACATGTATACTGTTGGTATAGATTTTGATTCTCGTGCTTATTTTACTGCTGCT

>Haplotype 8 (KX226376)

ACTAGTTGAACTATTTACCCTCCTTTATCTACTTCTGGTCATCCCGGTTCTAGTGTAGATTTGGCTATTTTTAGTTTGCACCTTTCTGGTATTAGTTCAATTTTAGGTGGTATTAATTTTATGTGTACTATTAAGAATTTGCGCTCTAGTTCTGTCTCTCTTAATAATATAAGTTTGTTTATTTGAACTATTTTTGTTACTGTTTTTTTATTGGTTTTATCTTTACCTGTTTTAGCTGGTGCTATTACTATATTATTGATTGATCGTAATTTTAATGGTTCTTTTTTTGATCCTAGTTTCGGTGGTAATCCTTTAATTTATCAACATTTGTTTTGGTTTTTCGGTCATCCAGAAGTTTATATTTTAATTTTACCTGCTTTTGGTATTATTAGTCAGTGTACTTTATATTTGACTGGTAAAAAAGAAGTTTTTGGTTATTTAGGAATGGTTTACGCTATCTTAAGTATTGGATTGATTGGTTGTGTGGTTTGGGCTCATCATATGTATACTGTTGGAATGGATTTTGATTCTCGTGCTTATTTTACTGCTGCA

>Haplotype 9 (KX226377)

ACTAGTTGAACTATTTATCCTCCTTTATCTACTTCTGGTCATCCTGGTTCTAGTGTAGATTTGGCTATTTTTAGTTTGCACCTTTCTGGTATTAGTTCAATTTTAGGTGGTATTAATTTTATGTGTACTATTAAGAATTTGCGCTCTAGTTCTGTCTCTCTTAATAATATAAGTTTGTTTATTTGAACTATTTTTGTTACTGTTTTTTTATTGGTTTTATCTTTACCTGTTTTAGCTGGTGCTATTACTATATTATTGATTGATCGTAATTTTAATGGTTCTTTTTTTGATCCTAGTTTCGGTGGTAATCCTTTAATTTATCAACATTTGTTTTGGTTTTTCGGTCATCCAGAAGTTTATATTTTAATTTTACCTGCTTTTGGTATTATTAGTCAGTGTACTTTATATTTGACTGGTAAAAAAGAAGTTTTTGGTTATTTAGGAATGGTTTACGCTATCTTAAGTATTGGATTGATTGGTTGTGTAGTTTGGGCTCATCATATGTATACTGTTGGAATGGATTTTGATTCTCGTGCTTATTTTACTGCTGCA

>Haplotype 10 (KX226378)

ACTAGTTGGACTATTTATCCTCCTTTATCTACTTCTGGTCATCCTGGTTCTAGTGTAGATTTGGCTATTTTTAGTTTGCACCTTTCTGGTATTAGTTCAATTTTAGGTGGTATTAATTTTATGTGTACTATTAAGAATTTGCGCTCTAGTTCTGTTTCTCTTAATAACATAAGTTTGTTTATTTGAACTATTTTTGTTACTGTTTTTTTATTGGTTTTATCTTTACCTGTTTTAGCTGGTGCTATTACTATATTGTTGATTGATCGTAATTTTAATGGTTCTTTTTTTGATCCTAGTTTCGGTGGTAATCCTTTAATTTATCAACATTTGTTTTGGTTTTTTGGTCATCCGGAAGTTTATATTTTAATTTTGCCTGCTTTTGGTATTATTAGTCAGTGTACTTTATATTTGACTGGTAAAAAAGAAGTTTTTGGTTATTTAGGAATGGTTTACGCTATTTTAAGTATTGGATTAATTGGTTGTGTAGTTTGAGCTCATCATATGTATACTGTTGGAATGGATTTCGATTCTCGTGCTTATTTTACTGCTGCA

>Haplotype 11 (KX226379)

ACTAGTTGGACTATTTATCCTCCTTTATCTACCTCTGGTCATCCTGGTTCTAGTGTAGATTTGGCTATTTTTAGTTTGCACCTTTCTGGTATTAGTTCAATTTTAGGTGGTATTAATTTTATGTGTACTATTAAGAATTTGCGTTCTAGTTCTGTTTCTCTTAATAATATAAGTTTGTTTATTTGGACTATTTTTGTTACTGTTTTTTTATTGGTTTTATCTTTACCTGTTTTAGCTGGTGCTATTACTATATTATTGATTGATCGTAATTTTAATGGTTCTTTTTTTGATCCTAGTTTCGGTGGTAATCCTTTAATTTATCAACATTTGTTTTGGTTTTTTGGTCATCCGGAAGTTTATATTTTAATTTTACCTGCTTTTGGTATTATTAGTCAGTGTACTTTATATTTGACTGGTAAAAAAGAAGTTTTTGGTTATTTAGGAATGGTTTATGCTATTTTAAGTATTGGGTTAATTGGTTGTGTAGTTTGAGCTCATCATATGTATACTGTTGGAATGGATTTTGATTCTCGTGCTTATTTTACTGCTGCA

>Haplotype 12 (KX226380)

ACTAGTTGGACTATTTACCCTCCTTTATCTACTTCAGGTCATCCAGGTTCTAGTGTTGATTTGGCTATTTTTAGTTTACATCTTTCTGGTATTAGTTCTATTTTAGGTGGTATTAATTTTATGTGTACTATTAAAAATTTGCGTTCTAGTTCTGTTTCTCTTAATAATATAAGTTTATTTATTTGAACTATTTTTGTTACTGTCTTTTTATTGGTTTTGTCTTTACCTGTTTTAGCAGGTGCTATTACTATGTTATTGATTGATCGTAATTTTAATGGTTCTTTTTTTGATCCTAGTTTTGGTGGTAATCCTTTGATTTATCAGCATTTGTTTTGGTTTTTTGGTCATCCGGAAGTTTATATTTTAATTTTGCCTGCTTTTGGTATTATTAGTCAGTGTACTTTATATTTGACTGGTAAGAAAGAGGTTTTTGGTTATTTAGGTATGGTTTATGCTATTTTAAGTATTGGTTTAATTGGTTGTGTAGTTTGAGCTCATCATATGTATACTGTTGGTATGGATTTCGATTCTCGTGCTTATTTCACTGCTGCC

>Haplotype 13 (KX226381)

ACTAGTTGGACTATTTATCCTCCTTTATCTACTTCAGGTCATCCAGGCTCTAGTGTTGATTTGGCTATTTTTAGTTTACATCTTTCTGGTATTAGTTCTATTTTAGGTGGTATTAATTTTATGTGTACTATTAAAAATTTGCGTTCTAGTTCTGTTTCTCTTAATAATATAAGTTTATTTATTTGAACTATTTTTGTTACTGTCTTTTTATTGGTTTTGTCTTTACCTGTTTTAGCAGGTGCTATTACTATGTTATTGATTGATCGTAATTTTAATGGTTCTTTTTTTGATCCTAGTTTTGGTGGTAATCCTCTGATTTATCAGCATTTGTTTTGGTTTTTTGGTCATCCAGAAGTTTATATTTTAATTTTACCTGCTTTTGGTATTATTAGTCAGTGTACTTTATATTTGACTGGTAAGAAAGAGGTTTTTGGTTATTTAGGTATGGTTTATGCTATTTTAAGTATTGGTTTAATTGGTTGTGTAGTTTGAGCTCATCATATGTATACTGTTGGTATGGATTTCGATTCTCGTGCTTATTTTACTGCTGCC

>Haplotype 14 (KX226382)

ACTAGTTGGACTATTTATCCTCCTTTATCTACTTCAGGTCATCCAGGTTCTAGTGTTGATTTGGCTATTTTTAGTTTACATCTTTCTGGTATTAGTTCTATTTTAGGTGGTATTAATTTTATGTGTACTATTAAAAATTTGCGTTCTAGTTCTGTTTCTCTTAATAATATAAGTTTATTTATTTGAACTATTTTTGTTACTGTTTTTTTATTGGTTTTGTCTTTACCTGTTTTAGCAGGTGCTATTACTATGTTATTGATTGATCGTAATTTTAATGGTTCTTTTTTTGATCCTAGTTTTGGTGGTAATCCTTTGATTTATCAGCATTTGTTTTGGTTTTTTGGTCATCCAGAAGTTTATATTTTAATTTTGCCTGCTTTTGGTATTATTAGTCAGTGTACTTTATATTTAACTGGTAAGAAAGAGGTTTTTGGTTATTTAGGAATGGTTTATGCTATTTTAAGTATTGGTTTAATTGGTTGTGTAGTTTGAGCTCATCATATGTATACTGTTGGTATGGATTTTGATTCTCGTGCTTATTTCACTGCTGCC

>Haplotype 15 (KX226383)

ACTAGTTGGACTATTTATCCTCCTTTATCCACTTCAGGTCATCCAGGTTCTAGTGTTGATTTGGCTATTTTTAGTTTACATCTTTCTGGTATTAGTTCTATTTTAGGTGGTATTAATTTTATGTGTACTATTAAAAATTTGCGTTCTAGTTCTGTTTCTCTTAATAATATAAGTTTATTTATTTGAACTATTTTTGTTACTGTTTTTTTATTGGTTTTGTCTTTACCTGTTTTAGCAGGTGCTATTACTATGTTATTGATTGATCGTAATTTTAATGGTTCTTTTTTTGATCCTAGTTTTGGTGGTAATCCTTTGATTTATCAGCATTTGTTTTGGTTTTTTGGTCATCCAGAAGTTTATATTTTAATTTTGCCTGCTTTTGGTATTATTAGTCAGTGTACTTTATATTTGACTGGTAAGAAAGAAGTTTTTGGTTATTTAGGTATGGTTTATGCTATTTTAAGTATTGGTTTAATTGGTTGTGTAGTTTGAGCTCATCATATGTATACTGTTGGTATGGATTTTGATTCTCGTGCTTATTTCACTGCTGCC

>Haplotype 16 (KX226384)

ACTAGTTGGACTATTTATCCTCCTTTATCTACTTCAGGTCATCCGGGTTCTAGTGTTGATTTGGCTATTTTTAGTTTACATCTTTCTGGTATTAGTTCTATTTTAGGTGGTATTAATTTTATGTGTACTATTAAAAATTTGCGTTCTAGTTCTGTTTCTCTTAATAATATAAGTTTATTTATTTGAACTATTTTTGTTACTGTTTTTTTATTGGTTTTGTCTTTACCTGTTTTAGCAGGTGCTATTACTATATTATTGATTGATCGTAATTTTAATGGTTCTTTTTTTGATCCTAGTTTTGGTGGTAATCCTTTGATTTATCAGCATTTGTTTTGGTTTTTTGGTCATCCAGAAGTTTATATTTTAATTTTGCCTGCTTTTGGTATTATTAGTCAGTGTACTTTATATTTGACTGGTAAGAAAGAGGTTTTTGGTTATTTGGGTATGGTTTATGCTATTTTAAGTATTGGTTTAATTGGTTGTGTAGTTTGAGCTCATCATATGTATACTGTTGGTATGGATTTCGATTCTCGTGCTTATTTTACTGCTGCC

>Haplotype 17 (KY548505)

ACTAGTTGAACTATTTATCCTCCTTTATCTACTTCTGGTCATCCCGGTTCTAGTGTAGATTTGGCTATTTTTAGTTTGCACCTTTCTGGTATTAGTTCAATTTTAGGTGGTATTAATTTTATGTGTACTATTAAGAATTTGCGCTCTAGTTCTATCTCTCTTAATAATATAAGTTTGTTTATTTGAACTATTTTTGTTACTGTTTTTTTATTGGTTTTATCTTTACCTGTTTTAGCTGGTGCTATTACTATATTATTGATTGATCGTAATTTTAATGGTTCTTTTTTTGATCCTAGTTTCGGTGGTAATCCTTTAATTTATCAACATTTGTTTTGGTTTTTTGGTCATCCAGAAGTTTATATTTTAATTTTACCTGCTTTTGGTATTATTAGTCAGTGTACTTTATATTTGACTGGTAAAAAAGAAGTTTTTGGTTATTTAGGAATGGTTTATGCTATCTTAAGTATTGGATTGATTGGTTGTGTAGTTTGGGCTCATCATATGTATACTGTTGGAATGGATTTTGATTCTCGTGCTTATTTTACTGCTGCA

*Haplotypes 2 and 3 were found in humans and dogs. Therefore they have two different GenBank entries.

**Single copy molecular genetic markers**

*>ytP274*

CAGGACCACCTGGACAAGTTGTATATGTAGAACCGGATGCTAATAGAATTGAACCAATATCAGGGCCACCAGGACCACAAGGCCCCCCAGGACCTCAAGGACCACCAGGAATTCAAGGACCTAGAGGTGAACCAGGAATAGGAATGCCAGGACCGCCGGGATTATTTACAGGTTTAACAGAAATGGATCTTGCAAGGATAGCTGCATATCCAGGAATCAAAGGTGAATGTGTTGAAAGAAAGGGAGTGGATGATTATAGCAATGATTCAGAAGAGCTTCCTATATATGATAGAAAGATTCACAGACAAAACAGTAAAGGAGAAAAAGGAGATAAAGGTGATCCTGGACCACAAGGACCACCAGGTCTACCAGGACTTTCAGGAACAACAAAAACATCTGCTACCTACACAGCTCCAGGAGGCGTAGAAGTTTACCAAACAACACAAGAATTATTGGGAGCTACAAATTCTTTTAGACAAGGTGCATTGGCTTTCAGTATTTCATCCCAACAATTAATGATCAGAGTGGCATCAGGATGGAAAG

*>ytP289*

TGAAACAGGAAAACACATCTACTGATACTATAACAGGAATAAAAGTTGTAATTGTTAATAATAATTTATTAAATCCAATTTTATATATTATGTCAAAAAGTATTGTCCTTGTTTATGATATAAAAAGAGATTCATTTGATAGTTATATTGATAATGAAGATTATGAAATGGTTCAAGTTTGGGACTTTAATCCATCTTTAAATTCAATTACTTTTGCATCAAAAGATATTGTTCACAAAATTTCTGTCAATTCTAATCATATGTGTGCATCGATTAAATATGATAGTATTCATAAAGGTATTGATAAGCTACAAATAATTCATGTTGGAAAAAGTATTGCTGTGTTATGGAAATCATCACATAGTTTAATTAATCTTACAATTTATGATACTGAATGTAATATTAATACATACCACCATCGTCAACTTCCTTTGAAGTCTTTTATTTTTACAATTAGTGGAATTTTATACCTTGTAACTAGTGATGGAAGATTGATTAGTTTAATTGAAAGTACAACTGAACAAAAAATGGATTTACTTTTAATTAAAAATATGTTTGATGTAGCTATTCAACTTGGTGAAGCTAATGGTATGTCATTATCACATGTACATAAAAAGTATGCGATTTATTTATTAGCCAAAAATGACTATGATAATGCACTTAAACATTTTAAAAAAGCAATTGGAGATGTTGAAACTTCATTTGTTATTAAAAGTTATTTAGAAGGTTCAAAATTAATGTTACTGCGTGAATATCTTGAACACT

*>ytP290*

TGCTGCCTCAACAATGTACAAGATAATTCAATTAAACAAAGATTACTTATACATTATTTAGGTCCTAAAGCTTTTGATCAATTATATATAATGTTATATCCCAAGTGCCTTTTTAATATGCCTTTTGATGAATTTTTATCAAGCTGTACAGGATGCTTTGGAAATGATATATCAAATGAAAATTATAACTCTTCATACCCTTATTGTTATTCAATTAATGATTTTATTAATCTTAAACAATCATCAAATGAAAGTATCTCTGAATTTTACTTATTACTGAAACAATCCGCCATTAATTTGGGATTAAACGATTCTGAACTTCATCAGAAAATTATGTATCGCACATTCATGAATGGATTATACAATCTAGAGATTAGAAAACGGTTAAAAAAAGAAAAACAAGTTATTAAAAGCCTTTTAGATGCCTATAA

Polymorphic positions are under laid in red.
